# Assessing Self-Help Orientation Among German Rehabilitation Clinics: Website Content Analysis

**DOI:** 10.2196/67428

**Published:** 2025-11-03

**Authors:** Elâ Ziegler, Thea Bartzsch, Sabine Bütow, Alf Trojan, Ines Krahn, Daniel Lüdecke, Nicole Usko, Christopher Kofahl

**Affiliations:** 1 Institute of Medical Sociology, University Medical Center Hamburg-Eppendorf, Martinistr. 52, Hamburg, 20246, Germany, 49 40-7410-53396; 2 German Working Group Self-Help Groups e. V., Gießen, Germany; 3 Network for Self-Help Friendliness and Patient Orientation in Health Care, Berlin, Germany

**Keywords:** patient centeredness, peer support, self-help groups, rehabilitation, website analysis, self-help, orientation, Germany, rehabilitation clinics, content analysis, health care, logistic regression, medical websites

## Abstract

**Background:**

Patient-centeredness has become a guiding principle of delivering quality health care. Integrating self-help services in health care facilities through collaboration is a vital part of this, specifically in rehabilitation. Despite increasing efforts to promote cooperation between rehabilitation clinics and self-help groups and organizations in Germany, implementation remains inconsistent, and research on this is particularly limited.

**Objectives:**

This study sought to examine the “self-help friendliness” (SHF) of rehabilitation clinics, considering the significance of self-help in their internet presence as a central source of patient information. The research objectives are thus to measure and compare the self-help orientation among rehabilitation clinics’ websites as an indicator of SHF to assess which clinic criteria are associated with self-help presentation on the clinic websites.

**Methods:**

A quantitative content analysis of 400 randomly chosen rehabilitation clinic websites was conducted as part of the KoReS project (self-help friendliness and cooperation with self-help among rehabilitation clinics in Germany) that is co-designed, conducted, and disseminated in collaboration with public health and patient representatives. Websites were systematically screened using a newly developed 16-criteria coding instrument assessing self-help orientation. A score was formed from these criteria ranging from 0 to 16 points. Univariate analyses describe the score distributions. Binomial logistic regression analyses were performed to determine the self-help orientation on the websites depending on characteristics of the rehabilitation clinics (size, indication area, and sponsorship).

**Results:**

Of the 400 clinics, 61.0% (n=242) scored low on self-help orientation, with the majority (41.8%; n=167) not being self-help oriented at all. Conversely, 39.5% (n=158) of the clinic websites demonstrated high self-help orientation, with 7.3% (n=29) of them achieving exceptional scores. Overall, a mean 4.4 (SD 4.1) of 16 points was reached and basic self-help orientation criteria were fulfilled by the clinics. Regression analysis revealed clinics covering the indication areas: oncology (odds ratio [OR] 2.64; *P*=.01), neurology (OR 2.73; *P*=.003) or addiction (OR 3.04; *P*<.001) to significantly predict higher self-help orientation scores. Facility size, sponsorship type, and the number of specialist indication areas did not impact the self-help orientation of the websites overall.

**Conclusions:**

This is the first analysis measuring the self-help orientation of rehabilitation clinic websites and indicates that it falls short of its potential. The findings suggest that greater emphasis on self-help display and collaboration with self-help in rehabilitation is needed. It can be achieved by using the concept of SHF, integrating self-help closely into clinic missions and treatment plans and considering the criteria developed in designing clinic websites to increase patient orientation.

## Introduction

Patient-centeredness has become a central aim of high-quality health care toward the needs and capabilities of patients [1,2]. One crucial element of patient-centeredness is the acknowledgment of the views of patients and their active involvement in integrated and comprehensive health care [3-5]. This includes collaboration with self-help groups and organizations to achieve patient involvement, patient empowerment, and continuity of care [3,5,6]. Self-help, understood as a proxy for peer support, mutual aid, patient support groups, self-help groups, and organizations, has been shown to positively impact patients in their empowerment, self-management, health literacy, and quality of life [2,7-10]. Subsequently, self-help is increasingly recognized as an important pillar of health care, particularly for patients with chronic diseases and disabilities [11-15]. In Germany, self-help has developed into a wide landscape of nearly 100,000 self-help groups, about 300 national self-help organizations, and more than 300 regional self-help clearinghouses for the support of self-help groups across the country [11]. More than half of the groups focus on chronic diseases or disabilities, and more than a third on addictions [11]. Their work comprises peer counseling and political patient advocacy to improve care quality and strengthen patient rights [13,16,17].

Due to the potential of self-help, there are growing efforts to develop and strengthen the cooperation between health care providers and self-help initiatives. One example is the concept of self-help friendliness (SHF) in health care and its quality criteria [15,18,19]. SHF is anchored in the German network “Self-Help Friendliness and Patient Orientation in Health Care” (SPiG), which was founded in 2009 [20]. SPiG consists of more than 400 members, such as patient umbrella organizations, self-help groups, rehabilitation clinics, and hospitals. Meanwhile, there are similar models in Austria and Switzerland [21,22]. SHF aims to initiate, increase, and improve cooperation between self-help representatives and health care providers in (1) hospitals, (2) rehabilitation clinics, (3) doctors’ offices, and (4) public health services. Health care facilities can apply for a certificate “self-help friendly hospital/rehabilitation clinic/medical practice” from the SPiG network if they fulfill the relevant conditions.

The concept of SHF is based on participatorily developed quality criteria for each of the 4 sectors that allow the implementation, evaluation, and assessment of a systematic cooperation between self-help groups and health care facilities. Although these quality criteria are very similar across the 4 sectors, there are small adaptations reflecting the specific conditions of each sector. In this study, we focus on the sector “inpatient rehabilitation.” For rehabilitation clinics, there are 5 quality criteria for SHF: (1) the clinic enables public relations of self-help in its facility. (2) Patients are provided opportunities for self-help participation. (3) In the clinic, a contact person for self-help affairs is appointed. (4) Clinic staff is qualified in self-help affairs. (5) The cooperation is consensually formalized, reliable, and sustainable.

Similar to the SHF concept, criteria for closer collaboration with self-help can also be found in quality management systems for various health care institutions, for instance, in the certification of rehabilitation clinics by the Federal Working Group for Rehabilitation (BAR) [19,20,23,24]. In the field of cancer treatment and care, comparable efforts are reflected in the quality criteria of the German Cancer Society, which require cooperation with self-help groups in cancer centers, approved with regular audits involving patient representatives. The same is true for the co-funding of the German comprehensive cancer centers by the German Cancer Aid [25,26]. In addition, the Federal Ministry of Health (BMG) calls for closer involvement of self-help in its National Cancer Plan [25], both in inpatient and outpatient care. Moreover, the Convention of the United Nations on the Rights of Persons with Disabilities demands in its Article 26 that all rehabilitation measures should include peer support [27]. In Germany, the acknowledgment of health-related self-help and the funding of its essential support structures (self-help clearing houses, allowances, and staff in offices of self-help organizations) is also anchored mainly in the statutory health insurance and pension insurance legislation as well as some further public domains [11,28].

Inpatient rehabilitation offers great potential for collaboration with self-help with high benefit, especially for patients with chronic conditions, such as cancer, addiction, or psychosomatic disorders [15,29]. Self-help groups can stabilize the success of rehabilitation, ease the transition, and enhance patient participation and empowerment [14,15,30]. Therefore, a collaboration between self-help and rehabilitation clinics is vital and should be an aim that the facilities and their funders strive for [31]. As one SHF quality criterion is to refer patients to self-help groups by informing them about self-help via the facility’s print and digital media [15,32,33], the internet is one key source of health information, support, and guidance for patients [34,35]. Thus, the homepages of rehabilitation clinics offer an opportunity for the provision of relevant self-help information [36-39], specifically as they are seen as a trusted source for health information [40]. They can guide patients, who may not be familiar with self-help, with reliable, preselected, and customized quality information on the topic [41].

The extent to which rehabilitation clinics engage in cooperation with self-help organizations, and the degree to which they fulfill SHF criteria, is difficult to determine due to the absence of empirical data to date. Therefore, this study systematically assesses the extent to which clinic websites are self-help oriented as an indicator of the SHF of the clinics’ public image. This investigation is one subproject of the project “cooperation between rehabilitation clinics and health-related self-help in Germany” (KoReS) [15], funded by the German Pension Insurance. The research objectives are thus to measure and compare the visibility of self-help among rehabilitation clinics’ websites. The aim is to get an empirically based impression of the significance of self-help in rehabilitation clinics’ self-expression. This also serves to identify patterns of clinics that are particularly committed to self-help in their external presentation.

## Methods

### Data Collection and Sampling

Depending on different existing lists, the number of rehabilitation clinics in Germany varies from around 1100 to 1400. The most reliable and weekly updated list is the list of inpatient rehabilitation clinics certified by the Federal Working Group for Rehabilitation (BAR) as per Section 37 (3) Social Code IX (SGB IX) [42]. We have chosen this data source because these clinics meet the requirements of an accredited internal quality management system that successfully implements quality criteria such as a participation-oriented mission statement and an indication-specific rehabilitation concept of the clinic [24]. The chosen list from March 22, 2024 contained 1146 clinics; however, 11 clinics in this list are located in Austria (having contracts with German social insurances). We have excluded them. This resulted in a total of 1135 clinics. As this was still too numerous for a full analysis with the given resources, we analyzed a sample of 400 clinic websites randomly selected by computer-generated numbers to represent one-third of all listed clinics in the database [42]. In the further course of the procedure, it turned out that 7 of the 400 selected clinics did not have a website. In these cases, the clinic with the previous number was included.

With this random sample, we conducted a systematic quantitative content analysis as defined by Riffe et al [43]. The websites were systematically screened for self-help references to quantify and evaluate the relevance of self-help in the public relations work of the facilities. The clinics cover a range of indications, including the 5 core indications of interest (Oncology, Neurology, Addiction, Psychosomatics, and Orthopedics) [15], treating a variety of patients. Background metadata of the clinic characteristics such as the provider, size, location, and indication area of the facilities was drawn from the list of hospitals and rehabilitation facilities of the Federal and State Statistical Office [44].

### Data Coding

Instruments that assess a self-help orientation of homepages of medical facilities are not available so far. Therefore, a coding sheet with relevant criteria assessing the self-help orientation of rehabilitation clinics and corresponding coding rules [43] was developed in an iterative participatory process from March to May 2024 by the research team and self-help representatives. The composition of the project team ensured a broad range of expertise, drawing on many years of self-help research, self-help practice, and patient involvement. This was particularly valuable, as 2 team members also served as patient representatives, allowing them to contribute the patient’s perspective. The development of the screening criteria was literature-based, considering SHF criteria [32,33] and general criteria for reliable patient information on the internet, such as actuality and content. Further criteria that are relevant for patients include relevance, findability, focus on the patient, and additional resources of information [40,45]. The criteria development started with a subsample of 20 clinic websites that were awarded the SHF quality seal by the SPiG network [15]. First, a qualitative analysis investigated if and how these self-help–friendly clinics addressed self-help–relevant topics on their homepages in a discursive process within the research team. Second, an initial pool of 26 criteria has been extracted from the qualitative analysis. Third, the item pool was refined, specified, and narrowed down in dialogue and consultation with the self-help and patient representatives and a pilot screening of the 20 homepages. For the following pretest, a randomly selected subset of 50 clinic websites was screened and deemed sufficiently large to cover all criteria to increase the validity and reliability of the criteria system [43] as the range of fulfilling hardly any criteria up to nearly all criteria was fully covered.

**Figure 1.**
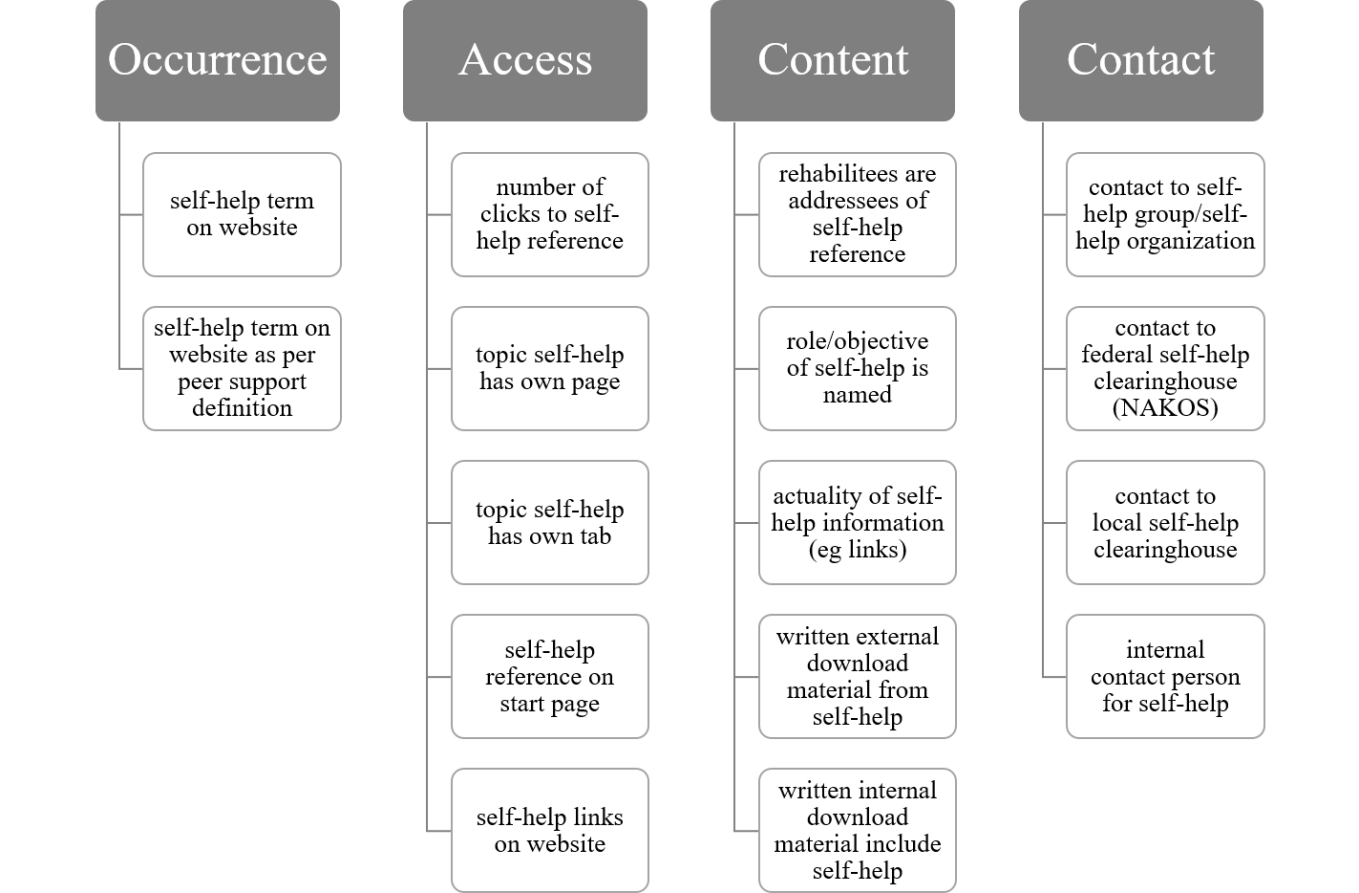
Self-help orientation screening domains and criteria for clinic websites.

After the pretest, ambiguous or unnecessary criteria were modified, revised, or removed until the criteria were clear and unambiguous. After retesting the criteria system with half of the sample, the process resulted in a 16-criteria assessment tool. These 16 criteria reflected adequately the depth and the qualities of the websites and met content validity and reliability. The final criteria were categorized into the following 4 dimensions: the occurrence of self-help on the websites (2 criteria), access to self-help references (5 criteria), content of self-help references (5 criteria), and contact information (4 criteria) (Figure 1). From the patients’ perspective, these criteria and dimensions serve to make them aware of self-help resources, to show them the purpose, significance, and potential benefits for them in coping with their illness, and to guide them toward further self-help support if they are willing to join a self-help group or organization, even after rehabilitation. For example, the clinic website contains the term “self-help” or its synonyms, the topic “self-help” has its own page or tab on the website, the information is up-to-date, references or contacts to self-help associations or clearinghouses are available and current, or contact persons for self-help affairs in the clinic are named.

The criteria were primarily assigned dichotomous values, that is, 0=“not available” (including unclear cases) and 1=“available.” In addition, the value 99 was allocated for “not applicable.” Thus, either 0 or 1 point could be achieved for each criterion except criterion 3 (number of clicks to self-help reference). This criterion was coded with 0 for “6 or more clicks,” 0.5 for “4‐5 clicks,” and 1 for “0‐3 clicks.” The websites were systematically screened for self-help orientation by a student coder based on the criteria fulfillment using a Microsoft Excel coding sheet (see Multimedia Appendix 1) closely monitored by the senior researchers. Since all websites were systematically coded by the same rater, interrater-reliability tests were not applicable. Nevertheless, even without cross-checking by a second or third rater, the rater reliability can be considered high, as each individual criterion is discrete and unambiguous in its meaning, because the decision as to whether it applies or not depends solely on whether the relevant information appears on the website or not. In other words, there is hardly any room for interpretation here. Furthermore, to avoid overlooking difficult-to-find information, the Google search operator “site:” was used to search for relevant self-help keywords and synonyms within a website as a whole, including subsections and downloadable documents. A very few remaining irregularities or uncertainties were addressed in regular team meetings with the senior researchers and resolved through discussion.

### Measures

#### Outcome

We created a self-help orientation score by summing points based on the 16 criteria. The individual websites received one point for each criterion met, resulting in a total score ranging from 0 (no self-help orientation) to 16 (extraordinarily high self-help orientation). The internal consistency of the scale is very high: The Cronbach α value of 0.902 suggests a steady increase in self-help orientation depending on the single item difficulty. The (unrotated) principal component analysis supports this 1-factor consistency as 14 of the 16 items have their highest loading on the first of 3 components, none on the second and 2 on the third (criteria 11 and 14). The latter items are the ones with the lowest agreement (3% and 6.5%); thus, this factor reflects the skewness or the difficulty of the items, respectively. The second component does not allow for a meaningful interpretation, even in the orthogonally and skewed rotated principal component analysis. All this points to one strong general factor.

The scale distribution, however, is positive skew (with 41.8% achieving only 0 to 1 points) and further shows 2 peaks at 5 and 7 points. The mean value is 4.35 (SD 4.107), median 4.5 (IQR=7), skewness=0.586 (SE=0.122), and kurtosis=−0.691 (SE=0.243). Based on this distribution and after having had a closer look at the achieved and the not achieved criteria, respectively, we categorized this score into 4 levels of website self-help orientation for further descriptive analyses: “not at all” (0‐1 points), “somewhat” (2‐5 points), “fully” (6‐11 points), and “exceptionally“ (12‐16 points). Since the distribution of this score does not meet the preconditions for multiple linear regression (Kolmogorov-Smirnov test=0.210, df=400; *P*<.001), we decided to use logistic regression models for further analyses and thus dichotomized the score into 2 groups: low/basic self-help orientation (scores ≤5) and high/advanced self-help orientation (scores >5). The low group contains the first 2 levels “not at all” and “somewhat,” and the high/advanced group contains the 2 levels “fully” and “exceptionally.” Fulfilling less than a third of the criteria was therefore deemed low.

#### Independent Variables

We examined 4 available clinic characteristics, sourced from the Federal and National Statistical Office’ lists of rehabilitation facilities [44,46], as predictors for being more or less self-help oriented. These clinic characteristics were

1. clinic sponsorship (statutory, nonprofit, or private) because there might be differences due to the economic models and cultures,
2. facility size (number of available beds) assuming that larger clinics have more capacity (specifically staff) to provide more measures such as self-help orientation,
3. coverage of specific indication areas (such as oncology or neurology) assuming that there is a different integration of self-help due to different conditions,
4. the breadth of specialization (covering exactly one area vs covering multiple areas) assuming that a higher number of indication areas would increase the chance of integrating self-help.

### Statistical Data Analyses and Variable Building

For data analysis, we used the R statistical software (R Core Team; version 4.4.2). We calculated frequency counts and means for univariate descriptive statistics. Logistic regressions were applied to determine how strongly the independent variables (clinic characteristics) were associated with self-help orientation. Model performance checks were run to test for observation independence, absence of multicollinearity, and lack of influential outliers [47]. Statistical significance was set to an α level of .05.

### Ethical Considerations

Ethical approval for the KoReS study was obtained from the University Medical Center Hamburg Local Psychological Ethics Committee at the Center for Psychosocial Medicine (approval number LPEK-0648) and conforms to the ethical guidelines of the Declaration of Helsinki. In this substudy, presented in this article, no human participants, medical records, patient information, observations of public behaviors, or secondary data analyses were involved or used.

The study aims to provide systematically and representatively gathered data from the homepages of rehabilitation clinics to show (1) whether these homepages contain self-help–related information at all, (2) how detailed this information is, (3) how prominent this information is embedded in the homepage, and (4) whether and how the homepage is connected to other self-help–related resources. Second, the data are used to analyze whether and which rehabilitation clinics’ characteristics are associated with self-help orientation on the homepage. As this research is unique so far, we had to develop our own coding and rating scheme for self-help orientation.

## Results

### Descriptive Analyses of Clinic Characteristics

Descriptive analysis revealed that the sample of 400 clinics represents the total population of n=1146 BAR-certified clinics well. Most clinics of the corresponding websites in the analysis were privately owned (n=214, 53.5%) and medium-sized with fewer than 300 available beds (88%) (Table 1). About one-fifth of the clinics were located in 2 of Germany’s largest federal states, Baden-Württemberg and Bavaria. The major indication areas were orthopedics (n=135, 24.5%) and psychosomatics (n=74, 13.4%), among other areas of specialization such as addictive or neurological disorders and cancer. Most clinics focused on one single indication group (n=227, 56.8%), followed by clinics addressing 2 indication groups (22%). A total of 34 clinics were not assigned any specific indication area in the Federal and National Statistical Offices’ lists [44]. Only 13 clinics were members of the SPiG network, of which 7 were SHF certified.

**Table 1.** Characteristics of the rehabilitation clinics (N=400).

Clinic characteristics	n (%) or mean (SD)
Size (beds available), mean (SD)	146.8 (114.4)
Size (beds available), n (%)	
<100	160 (41.9)
100 to <300	192 (50.3)
≥300	30 (7.5)
Sponsorship type, n (%)	
Statutory	83 (20.8)
Nonprofit	103 (25.8)
Private	214 (53.5)
Specialist indication area, n (%)	
Oncology	32 (5.8)
Neurology	56 (10.2)
Addiction	60 (10.9)
Psychosomatics	74 (13.4)
Orthopedics	135 (24.5)
Federal state, n (%)	
Baden-Württemberg	76 (19.0)
Bavaria	72 (18.0)
Berlin	5 (1.3)
Brandenburg	10 (2.5)
Bremen	3 (0.75)
Hamburg	2 (0.5)
Hesse	45 (11.3)
Mecklenburg-Vorpommern	20 (5.0)
Lower Saxony	42 (10.5)
North Rhine-Westphalia	52 (13.0)
Rhineland-Palatinate	15 (3.8)
Saarland	6 (1.5)
Saxony	17 (4.3)
Saxony-Anhalt	6 (1.5)
Schleswig-Holstein	19 (4.8)
Thuringia	10 (2.5)
SPiG^a^ membership, n (%)	
Yes	13 (3.3)
No	387 (96.8)
Number of specialist indication areas, mean (SD)	1.6 (1.0)
Number of specialist indication areas, n (%)	
1	227 (56.8)
2‐3	124 (31.0)
>3	15 (3.8)
Not applicable	34 (8.5)

a SPiG: Self-Help Friendliness and Patient Orientation in Health Care.

### Self-Help Orientation Scoring of the Clinics

#### Univariate Analyses

Self-help orientation scores among the sample clinics ranged from 0 to 15 points. No clinic website fulfilled all 16 criteria, while any single criterion was fulfilled by at least some clinics, with “written external download material from self-help exists” as the rarest (Table 2). The highest score of 15 points was achieved by only 1 clinic, while 113 clinics did not fulfill any criteria at all (0 points). On average, the clinic websites reached mean 4.4 (SD 4.1) points. Most clinics (42%) were classified as “not at all” self-help oriented, scoring 1 point or less. “Somewhat” self-help oriented (scoring 2‐5 points) was achieved by 19% of the clinic websites, while nearly a third (32%) was classified as “fully” self-help oriented, scoring more than 5 to under 12 points. The highest level of being “exceptionally” self-help oriented, scoring ≥12 points, was reached by 29 clinics (7%).

**Table 2.** Self-help orientation screening criteria fulfillment of rehabilitation clinics (N=400).

Criteria	Fulfillment, n (%)
Occurrence	
Self-help term occurs on the website	287 (71.8)
Self-help term occurs on the website defined as peer support	233 (58.3)
Access	
Low number (≤3) of clicks until self-help reference occurs	186 (46.5)
Topic “self-help” has its own page	42 (10.5)
Topic “self-help” has its own tab	35 (8.8)
Self-help reference is made on the start page	52 (13.0)
Links to self-help pages exist	70 (17.5)
Content	
Rehabilitees are the addressees of the self-help reference made	185 (46.3)
The role/objective of self-help is stated	92 (23.0)
Actuality of the self-help information (eg, links) is given	215 (53.8)
Written external download material from self-help exists	12 (3.0)
Written internal clinic download material includes self-help	63 (15.8)
Contact	
Provision of contact to	
Self-help initiatives	114 (28.5)
The federal self-help clearinghouse (NAKOS)	26 (6.5)
Local/regional self-help clearinghouses	37 (9.3)
Clinic staff member for self-help affairs	77 (19.3)

Regarding the individual criteria fulfillment (Table 2), the univariate analysis revealed the first 2 basic criteria of the dimension “occurrence” to be covered the most by the websites (58.3%; n=233). The “access” dimension criterion “low number (≤3) of clicks until self-help reference occurs” was met by 46.5% (n=186) of the clinic websites, while the other criteria of this dimension were reached by only a maximum of 17.5% (n=70) of the clinics. For instance, 70 clinic websites provided links to integrated pages with self-help information, and in only 35 cases, the topic of self-help had its own prominent tab on the clinic website. Criteria fulfillment relating to the “content” of the self-help references varied between 3% (n=12) and 53.8% (n=215) of the clinics, with criterion 11 “external download material from self-help initiatives exists” being the rarest, as it was only provided by 12 clinics. Criteria focusing on the “contact” dimension and containing referrals to further contacts were achieved by a maximum of 28.5% (n=114) of the clinic websites.

#### Multivariate Analyses

Five multiple binary logistic regression analyses were performed for the core indications of interest. The regression analyses assessed what clinic characteristics predict low versus high scoring of self-help orientation among the rehabilitation clinic websites. Four independent variables were entered into the final regression models; their odds ratios (ORs) can be found in Table 3. The performance check for the models revealed no concerns regarding the assumptions of logistic regressions [46], such as observation independence, absence of multicollinearity, and lack of strongly influential outliers. None of the models except the model with addiction as an indication area was statistically significant, and they resulted in a very small amount of explained variance, as indicated by Nagelkerke *R*^2^ (<0.1), while the Hosmer-Lemeshow test showed a good model fit (*P*>.05) for each model.

**Table 3.** Logistic regression model predicting website self-help orientation (n=350)^a^.

Indication	Addiction		Oncology		Orthopedics		Psychosomatics		Neurology	
Independent variables	OR^b^ (95% CI)	*P* value	OR (95% CI)	*P* value	OR (95% CI)	*P* value	OR (95% CI)	*P* value	OR (95% CI)	*P* value
Nonprofit sponsor (Reference: statutory)	0.50 (0.25-1.00)	.049	0.69 (0.36-1.33)	.27	0.66 (0.34-1.26)	.21	0.68 (0.36-1.31)	.25	0.62 (0.32-1.19)	.15
Private sponsor (Reference: statutory)	0.82 (0.47-1.43)	.48	0.88 (0.51-1.54)	.66	0.88 (0.51-1.53)	.65	0.93 (0.53-1.63)	.80	0.80 (0.45-1.39)	.43
Facility size (beds)	1.00 (1.00-1.00)	.25	1.00 (1.00-1.00)	.85	1.00 (1.00-1.00)	.44	1.00 (1.00-1.00)	.48	1.00 (1.00-1.00)	.92
One specialist indication area (Reference: multiple)	1.13 (0.66-1.95)	.66	1.25 (0.72-2.17)	.42	0.98 (0.54-1.76)	.94	1.12 (0.65-1.92)	.69	1.48 (0.83-2.61)	.18
Addiction (Reference: No addiction)	3.04 (1.60-.77)	<.001	—^c^	—	—	—	—	—	—	—
Oncology (Reference: No oncology)	—	—	2.64 (1.22-5.72)	.01	—	—	—	—	—	—
Orthopedics (Reference: No orthopedics)	—	—	—	—	0.66 (0.38-1.16)	.15	—	—	—	—
Psychosomatics (Reference: No psychosomatic	—	—	—	—	—	—	0.55 (0.31-0.96)	.04	—	—
Neurology (Reference: No neurology)	—	—	—	—	—	—	—	—	2.73 (1.42-5.27)	.003
Nagelkerke *R*^2^	0.05	—	0.03	—	0.01	—	0.02	—	0.04	—

a All variables entered into the model.

b OR: odds ratio.

c Not applicable.

Concerning the clinics covering oncology, addiction, or neurology as an indication area, the regression analyses showed that the indication area variable significantly contributed to predicting higher self-help orientation scores. For instance, being specialized in cancer increased the likelihood of achieving higher self-help orientation scores significantly. Cancer rehabilitation clinics had 2.6 times higher odds of having a self-help–oriented website compared to all other clinics (OR 2.64, 95% CI 1.22-5.72; *P*=.01). Clinics specialized in addiction (OR 3.04, 95% CI 1.60-5.77; *P*<.001) or neurology (OR 2.73, 95% CI 1.42-5.27; *P*=.003) achieve even higher odds toward self-help orientation. In contrast, psychosomatics as an indication significantly decreased the likelihood for self-help orientation (OR 0.55, 95% CI 0.31-0.96; *P*=.04). Concerning the sponsorship of the clinics, the results demonstrate that lower self-help orientation scores were more likely to be achieved in clinics with nonprofit ownership for the indication area of addiction (OR 0.50, 95% CI 0.25-1.00; *P*=.049) compared to statutory-owned facilities. For the other indications, however, no statistical differences were found for the type of sponsorship. Other clinic characteristics, such as size and the number of specialized indication areas, were not consistently associated with the outcome and were not statistically significant. Furthermore, for the model with the indication area “orthopedics,” none of the variables significantly predicted a self-help orientation of the clinic websites.

## Discussion

### Principal Findings and Context to Prior Research

This is the first study to measure self-help orientation within German rehabilitation clinics’ websites. The websites were coded for the nature and extent of self-help content to determine the presence and awareness of and collaboration with self-help groups, self-help organizations, and self-help clearinghouses as an important indicator of patient-centered care. The study revealed that most clinic websites scored only moderately on self-help orientation, suggesting that most rehabilitation clinics do not visibly cooperate with self-help associations. In fact, more than 40% of the clinic websites were found to be not self-help oriented at all, as they did not contain any note about self-help. Although most criteria indicating self-help orientation are basic and easy to fulfill, on average, only a fourth of the given criteria were realized on the clinic websites. Moreover, the individual clinics’ self-help orientation was very heterogeneous, illustrated by the wide range of score with values from 0 to 15, respectively. Most websites, which contain at least some content about self-help, only provide basic information, such as mentioning self-help for rehabilitation patients or providing up-to-date information, such as links on the topic. More advanced criteria, such as internal or external download material about self-help, prominent positioning of the topic, and specific contact referrals for self-help, were only achieved by less than a fifth of the clinics. This number could be higher, as nearly half of the clinics were statutorily owned (eg, funded by the German Pension Insurance Federation) or nonprofit owned, and would therefore propose a more advanced interaction with self-help initiatives based on legislative regulations [11,28] and criteria of quality management systems [23].

References to additional resources, such as clearinghouses as interfaces between health care facilities and self-help groups, were extraordinarily rare. Self-help clearinghouses can play an important role in supporting rehabilitees upon returning home from their (often distant) rehabilitation stays to find suitable self-help groups within their local area.

As expected, the self-help orientation on the websites of the 13 SPiG member clinics and the SHF-certified clinics showed a particularly high self-help orientation. It is noteworthy, however, that quite a lot of clinics that are not part of the SPiG network or the SHF concept achieve a high self-help orientation score. The existing positive examples within or beyond the SHF framework show that most other clinic websites could provide more relevant self-help information than they currently do. Due to the potential of clinic homepages as a source of trustworthy information [45], it seems advisable to invest in the concept, structure, and content of the clinic’s website. This would also be helpful for rehabilitation staff to encourage their patients to use the clinic’s websites for further complementary information [45]. In contrast, most clinic websites perform well in presenting current information that focuses on the patient and their needs, which was also identified as important for high-quality patient information by other authors [40,45]. Here, SPiG membership can be useful to boost self-help orientation, as bivariate analysis (data not shown) revealed a significant positive correlation between membership and self-help orientation, even though only a few member clinics were included. Since the developed criteria allowed self-help orientation to be quantifiable, they can serve funders of rehabilitation clinics or the SPiG network to assess whether this specific part of patient orientation is being achieved.

Even when adjusting for clinic size, main indication, and number of specialist indication areas in multivariate analyses, funding type overall was not associated with higher self-help orientation. There is no obvious reason to assume that clinics under statutory, nonprofit sponsorship or funding by the German Pension Insurance Federation would consider a stronger self-help orientation that is associated with higher scores than privately-owned clinics. Hypothetically, higher scores would have been expected due to the sponsors’ legislation and commitment-based obligations mentioned above. As self-help orientation scores were overall low, rehabilitation facilities and their funding bodies should consequently recognize the value of self-help more strongly, to implement greater collaboration as part of patient-centeredness, in line with Fisher’s suggestion [14]. Furthermore, the funders should monitor the actual collaboration more rigorously. This result is in accordance with other research, which found that clinics do not sufficiently inform about self-help [48]. Thus, there is room for improvement in cooperation between self-help groups and organizations and inpatient rehabilitation clinics [48]. The need for more patient-centered rehabilitative care is also highlighted as particularly important for patients with chronic diseases and disabilities [4]. A more advanced collaboration would not only benefit the patients but may also aid in reducing costs for the health care system [14].

Our regression analyses also revealed that the type of indication focus is associated with self-help orientation, regardless of clinic size, organization, and number of specialist indication areas. This finding proposes that certain clinical conditions, such as oncological, neurological, and addictive diseases, are more likely to be connected with self-help and that those are considered in particular relevant for supportive self-help services because neurological, oncological, and addictive diseases usually have far-reaching consequences that affect the everyday life of patients on several levels. Subsequently, rehabilitation staff may regard participation in peer support as especially relevant for patients with these chronic conditions in contrast to orthopedic diseases with less extensive consequences. It also responds to patients with chronic diseases using the internet, such as clinic homepages, more frequently for information, as stated by Zhang et al [36]. Moreover, this result may reflect that self-help is particularly advanced, well-known, and accepted in certain indication areas [14]. After the mental health movement, peer support has expanded for other common chronic illnesses, such as cancer [49]. In Germany, this resulted in a dense network with cancer self-help groups widely available across Germany [11,17]. For chronic diseases with highly developed, organized self-help, such as addiction or cancer, more research exists, and based on this, self-help is more closely integrated into treatment standards and health policy [14,25,49,50]. This could make it easier for clinics to cooperate with self-help.

The regression model on the indication area addiction was the only significant model, and there was a clear association with higher self-help orientation scores. This may also be due to the existing established structures for addiction. Further, self-help for addiction is widely accepted and integrated into care in general, but also in rehabilitation [51], since the focus on addiction-related self-help has already been promoted since the early 20th century, initially by temperance organizations such as the Good Templars, and particularly since 1935 with the founding and subsequent global expansion of Alcoholics Anonymous [52].

Surprisingly, the results demonstrated that clinics focusing on psychosomatic disorders hold lower self-help orientation, although self-help originated in the field of mental health and particularly psychiatry [53]. Besides, the prevalence of mental health and psychosocial issues is often higher among patients with chronic conditions [14], so integrating self-help services targeting the mental health of patients is crucial. Höflich and colleagues [51] already illustrated low use of self-help groups after inpatient rehabilitation treatment. Instead, psychotherapy is more common in further outpatient care [51]. However, participation in self-help groups could potentially bridge the often long waiting times [54] until the start of outpatient psychotherapy. Therefore, more information should be provided on psychosomatic self-help groups in rehabilitation facilities to aid in stabilizing the rehabilitation process long term.

As self-help orientation did not significantly correlate with clinic size in the multivariate analyses, it might mean that limited resources are not so decisive for implementing cooperation with self-help as an indicator of patient-centeredness. Instead, existing effective facility structures could be more influential in implementing self-help friendliness. Previous literature has demonstrated that central contact persons in the health care facilities are a key factor for implementing collaboration with self-help groups [55]. Besides, integrating self-help into quality management, disease management programs, or mission statements of the clinics can be beneficial, as highlighted by other authors [48,49,55]. Notably, the explained variance was overall low in the regression models. Therefore, the findings emphasize system- and facility-related factors other than the ones assessed to be more relevant for implementing self-help orientation on the clinic websites. These factors might comprise the knowledge, awareness, attitudes, and individual motivation of the clinic management toward self-help, consistent with earlier studies [20,48]. Thus, using existing structures more effectively and promoting integrated care models is recommended to increase patient-centeredness. Although the results from this study are not generalizable to other countries, those with similarly defined self-help organization structures could still benefit from cooperation between self-help and medical facilities as described in this paper.

### Limitations and Future Research Implications

There are some methodological and conceptual limitations to consider in this research. First, websites were retrieved and analyzed for only 1 month. As online data are dynamic and websites can easily change over time, the findings are just a snapshot showing a point prevalence neglecting possible changes before and after the analysis. Future research could consequently consider tracking changes over time. Second, the reliability of the coding may be limited since there was only one active coder. However, the coding instrument was pretested and retested, its criteria are very clear, and the coding was carried out in constant exchange with 3 other researchers. Therefore, it can be expected that interrater reliability would have been high if tested with multiple coders, specifically, as there was limited scope for subjective assessments due to the clear allocation. Third, the indicators for the self-help orientation value were not weighted, although there are likely to be differences in their significance for patients. Since the significance is difficult to quantify, it might be helpful to consult patients or patient representatives for further validation of the instrument.

Another aspect to consider is that other relevant dimensions of patient-centeredness were not measured, as this study solely focused on collective self-help. For rehabilitation clinic websites, further criteria are important to cover a full and comprehensive patient-centeredness. These include the website usability, the readability, and simple language of the content, as well as the visual presentation such as the choice of colors so that the information can be processed by patients with different types of cognitive or visual impairments. Although this was not the focus of this study, it is recommended to expand the current category system to assess further dimensions of patient-centeredness and allow for achieving an even higher level of patient-centeredness based on future research. Potential future studies could additionally investigate how often rehabilitation staff advise patients to consult the facilities’ websites and to what extent they follow this advice. Furthermore, these results cannot claim that the contents found on the rehabilitation clinics’ websites represent the self-help–oriented resources and activities in the clinics themselves, and neither the actual patient experiences with self-help services, nor the real-world accessibility and usefulness of self-help groups for the patients. Hypothetically, we assume a significant correlation between a clinic’s engagement or nonengagement in promoting self-help and its reflection on the clinic’s homepage. Nevertheless, we cannot rule out the possibility that a self-help–friendly clinic may not feature its SHF on its website for a variety of reasons, for example, that no one has taken care of it, or that other aspects such as treatment concepts, clinic facilities, and surroundings, etc, have been given priority. Finally, including further relevant covariates may have increased the explained variance of this research. However, for this preliminary explorative study, the most relevant ones were considered, but for future research, more factors might be taken into account.

### Conclusions

This initial assessment of the self-help orientation level of rehabilitation clinic websites demonstrated that self-help orientation is only moderately developed. Thus, there is room for improvement for the clinics to become more patient-centered, for instance, by providing accessible and relevant further self-help resources. Despite favorable legal and social framework conditions, greater cooperation between rehabilitation clinics and self-help associations is needed, which is also evident on the clinic websites. The website analysis reveals that external characteristics, such as the sponsor type or size of the clinics, are not associated with self-help orientation on their homepages. Instead, individual clinic-internal factors might boost higher levels of self-help orientation, for instance, anchoring the role of self-help and cooperation as an aim in the mission statement and integrating self-help more closely into treatment plans. Along with the involvement of the self-help friendliness concept and motivated clinic employees, these steps may promote the potential of self-help as one important part of patient-centeredness more strongly. In the future, it could be helpful to publish resources for creating a more self-help–oriented website for rehabilitation clinics, such as examples of best practices or step-by-step instructions. In addition, the importance of health care providers’ homepages as a central source of information for patients should be emphasized more strongly in the SHF concept.

The general lack of in-depth research specifically quantifying how often, or how well, self-help is represented across a broad spectrum of health care provider websites suggests that this area is still evolving. The effectiveness of self-help groups themselves is well-documented, making their improved visibility on health care provider websites a logical next step in patient-centered care.

## Supplementary material

10.2196/67428 Multimedia Appendix 1 Quality criteria coding sheet.

## Abbreviations

BAR: Federal Working Group for Rehabilitation
BMG: Federal Ministry of Health
KoReS: self-help friendliness and cooperation with self-help among rehabilitation clinics in Germany
SGB: Social Code
SHF: self-help friendliness
SPiG: Self-Help Friendliness and Patient Orientation in Health Care
